# Two Triterpenoids, ARM-2 and RA-5, From *Protorhus longifolia* Exhibit the Potential to Modulate Lipolysis and Lipogenesis in Cultured 3T3-L1 Adipocytes

**DOI:** 10.1155/2024/3972941

**Published:** 2024-10-17

**Authors:** Musawenkosi Ndlovu, June C. Serem, Megan J. Bester, Zeno Apostolides, Andrew R. Opoku, Rebamang A. Mosa

**Affiliations:** ^1^Department of Biochemistry, Genetics and Microbiology, University of Pretoria, Lynnwood Rd, Hatfield, Pretoria 0002, South Africa; ^2^Cochrane South Africa, South African Medical Research Council, Tygerberg 7505, South Africa; ^3^Department of Anatomy, University of Pretoria, Pretoria 0002, South Africa; ^4^Department of Biochemistry and Microbiology, University of Zululand, EMpangeni, KwaDlangezwa 3886, South Africa

**Keywords:** adipocytes, hepatocytes, hypertriglyceridemia, lipid accumulation, lipogenesis, lipolysis, obesity, triterpenoids

## Abstract

Triterpenoids have been identified as potential novel lipid-lowering drugs for the treatment of hypertriglyceridemia. This study investigated the potential antilipogenic and/or antilipolytic effects of two triterpenoids (ARM-2 and RA-5) isolated from the stem bark of *Protorhus longifolia* (Benrh.) Engl. Employing a combination of in silico predictions and in vitro assays, the interactions between these triterpenoids and key proteins involved in lipogenesis and lipolysis were investigated. In silico molecular docking analysis predicted a favourable binding affinity of both triterpenoids to PPAR*γ*, SREBP-1, and AMPK, with lower binding affinity to C/EBP*α*, pancreatic lipase, and hormone-sensitive lipase (HSL). Both triterpenoids exhibited in vitro inhibition of pancreatic lipase with K_i_ and IC_50_ values ranging from 28.7 to 52.9 *μ*M and 27.6 to 35.8 *μ*M, respectively. Total and neutral lipid accumulation in differentiated 3T3-L1 adipocytes and the oleic acid–induced HepG2 cell model was inhibited, with ARM-2 showing better inhibition than RA-5. In the HepG2 model, the inhibitory activity of the two triterpenoids (at 25 and 100 *μ*M) was comparable to 50 *μ*M lovastatin, although the latter was cytotoxic, whereas both ARM-2 and RA-2 lacked cytotoxicity. Associated gene expression was similar to the effect of simvastatin where the expression of SREBP-1, PPAR*γ*, C/EBP*α*, and HSL was reduced and that of AMPK was unchanged. In vitro studies confirmed that ARM-2 and RA-5 also inhibited adipocyte lipolysis, where the reduction in glycerol release by 25 and 100 *μ*M was similar to 50 *μ*M lovastatin and simvastatin. This study identifies that the triterpenoids, ARM-2 and RA-5, have the potential to modulate lipogenesis and lipolysis.

## 1. Introduction

Abnormally elevated triglyceride (TG) levels in the bloodstream, referred to as hypertriglyceridemia (HTG), are closely linked to obesity and significantly heighten the risk of atherosclerotic cardiovascular disease and Type 2 diabetes mellitus. The rising prevalence of HTG in recent years has been a global concern, as HTG affects not only the quality of life but also contributes to high healthcare costs and high mortality rates [1, 2]. In addition to genetic predispositions, the risk of developing HTG increases with age, increased body weight, and insulin resistance, with sedentary lifestyles and consumption of high-fat diets having a further contributing effect [3, 4].

The development of HTG is largely related to the excessive synthesis and secretion of triglyceride-rich lipoproteins (TRLs) such as chylomicron and very-low-density lipoproteins (VLDLs), along with impaired plasma TRL clearance [2]. Subsequently, excess TGs in the blood and carbohydrate metabolites stimulate lipogenesis and lead to the storage of excess fats in adipose tissue. Key transcription factors, including sterol regulatory binding elements (SREBP-1), PPAR*γ*, and C/EBP*α*, play pivotal roles in fatty acid and TG synthesis, contributing to adipocyte expansion, a hallmark of obesity. Modulating certain lipogenic genes, such as SREBP-1 and adenosine monophosphate (AMP)–activated protein kinase (AMPK), has shown promising therapeutic benefits in lowering elevated blood lipids [5].

While adipocyte lipolysis can help reduce intracellular lipid accumulation, this also results in the release of TGs into the bloodstream as free fatty acids (FFAs) and glycerol. The flux of FFA into the blood stimulates hepatic fatty acid uptake and subsequent de novo lipogenesis. Hepatic TGs can either be stored as cytosolic lipids or assembled into VLDLs. The TRLs are released into the bloodstream where, if not properly cleared, they contribute to HTG [6]. Hepatic lipid accumulation can also lead to fatty liver diseases such as nonalcoholic fatty liver disease.

Since a diet rich in TGs exacerbates the risk of HTG, inhibiting dietary TG digestion and absorption has been an attractive strategy for reducing circulating TG levels. Orlistat, a pharmacological agent that functions by competitively inhibiting pancreatic lipase (PNLIP), is currently among the primary treatments for HTG. Statins such as simvastatin and lovastatin primarily target cholesterol synthesis and manage high cholesterol levels and are also used for the management of HTG [7]. However, the clinical use of the current lipid-lowering agents is limited due to various adverse side effects, including loss of libido, sleep disturbances, nausea, and allergic reactions [8, 9]. Therefore, there is a need for new biologically derived compounds for the treatment and management of lipid disorders that are as effective as existing drugs but have fewer or no side effects with the further benefit of multitarget effects on HTG [10, 11].

Due to their wide array of biological effects, triterpenoids have emerged as promising candidates for use as potential pharmacological agents for treating metabolic disorders [12–14]. Our research group has previously demonstrated the hypolipidemic [14, 15] and hypoglycemic [16] potentials of 3*β*-hydroxylanosta-9,24-dien-21-oic acid (RA-5) and methyl-3*β*-hydroxylanosta-9,24-dien-21-oate (RA-3), the lanosterol triterpenes from the stem bark of *Protorhus longifolia* (Benrh.) Engl. The hypocholesterolemic effects of RA-5 and a newly isolated triterpenoid (3*α*,26-dihydroxytirucalla-7,24-dien-21-oic acid [ARM-2]) from *P. longifolia* stem bark have recently been reported, and their bioactivity was linked to the regulation of cholesterol synthesis and LDL uptake in HepG2 cells [17]. As a result, the current study has sought to further evaluate the antilipogenic and/or antilipolytic effects of both ARM-2 and RA-5.

## 2. Materials and Methods

### 2.1. Reagents

The reagents purchased from Sigma-Aldrich Co (St. Louis, MO, United States) were oleic acid (OA), lipase from porcine pancreas, crystal violet (CV), Oil Red O (ORO), Nile red (NR), Lipolysis Colorimetric Assay Kit (MAK211), orlistat, simvastatin, lovastatin, dexamethasone, rosiglitazone, isobutylmethylxanthine, and insulin. Mouse 3T3-L1 fibroblasts and human HepG2 hepatoma were purchased from the American Type Culture Collection (ATCC) (Manassas, VA, United States). Dulbecco's modified Eagle's medium (DMEM) and foetal calf serum (FCS) were purchased from SepSci (Johannesburg, RSA) and BioSom (Pretoria, RSA). Oligo primers were purchased from Inqaba Biotech (Pretoria, RSA). An SV Total RNA isolation kit, GoScript Reverse Transcription Kit, and GoTaq qPCR Master Mix were purchased from ANATECH (Johannesburg, RSA). Oligo primers were purchased from Inqaba Biotech. An SV Total RNA isolation kit, GoScript Reverse Transcription Kit, and GoTaq qPCR Master Mix were purchased from ANATECH (Gauteng, RSA).

### 2.2. Plant Extraction and Isolation of the Plant Compounds

**Figure 1:**
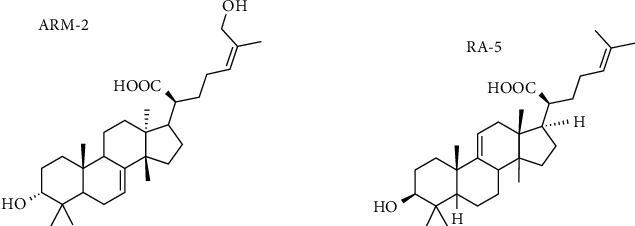
The chemical structures of 3*α*,26-dihydroxytirucalla-7,24-dien-21-oic acid (ARM-2) and 3*β*-hydroxylanosta-9,24-dien-21-oic acid (RA-5).

The plant triterpenoids ARM-2 and RA-5 were routinely isolated from the chloroform extract of *P. longifolia* stem bark (Voucher Specimen Number RA01UZ) following previously described protocols [17, 18]. The chemical structures of ARM-2 and RA-5 (Figure 1) were confirmed based on spectral (NMR, IR, and HRESIMS) data analysis and by comparison with literature data [17, 18].

### 2.3. Molecular Docking of the Compounds

The molecular docking of the compounds against PNLIP (2OXE), AMPK (5ISO, 4CFF), hormone-sensitive lipase (HSL) (3DNM), PPAR*γ* (5GTN), C/EBP*α* (1NWQ), and SREBP-1 (5GPD) was evaluated using Maestro from Schrodinger (2022_1). Three-dimensional crystal structures of the enzymes and proteins were obtained from the RCSB Protein Data Bank (PDB). While the SMILE notations of the reference standards were obtained from PubChem, those of the triterpenoids were generated after drawing their 2D structures in ACD/ChemSketch (freeware). The LigPrep function was used to prepare the ligands, while the protein preparation wizard was used to prepare the enzymes and proteins. Site mapping was performed to detect the catalytic sites of the proteins, followed by receptor grid generation. The structure–protein interactions were visually screened using Glide Extra Precision (XP), and a Glide score of −8.00 kcal/mol was set as the cut-off for better binding [19].

### 2.4. Construction of the Protein–Protein Interaction (PPI) Network

The Search Tool for the Retrieval of Interacting Genes/Proteins (STRING) database (https://string-db.org/) was used to construct a PPI network of several proteins involved in lipogenesis and lipolysis. The PPI network included the following proteins: SREBP-1c, PPAR*γ*, C/EBP*α*, AMPK, HSL, acetyl-CoA carboxylase (ACC) (ACACB), fatty acid synthase (FASN), and PNLIP. The resulting PPI network was subsequently visualized and analyzed using Cytoscape software Version 3.10.0 [17].

### 2.5. PNLIP Inhibition

The PNLIP inhibitory activity of the compounds was tested using p-nitrophenyl palmitate (p-NPP) as an artificial substrate [20]. The reaction mixture consisting of 0.061 M Tris-HCl buffer, pH 7.4, 200 U/mL porcine PNLIP, and the test compounds or orlistat (reference drug) (5–100 *μ*M) was preincubated at 37°C for 10 min. The reaction was initiated by adding 10 mM p-NPP at 37°C for 20 min. Then, the enzyme activity was determined calorimetrically by measuring the release of p-nitrophenol measured at 405 nm using a SpectraMax paradigm microplate reader (Molecular Devices Inc, San Jose, California). The IC_50_ values were determined using nonlinear regression from the absorbance versus concentration curves.

### 2.6. Enzyme Kinetics

The modes of enzyme inhibition were determined using the reaction protocol described above. While all the other parameters remained unchanged, the enzyme activity was measured at increasing substrate concentrations (2–10 mM) in the presence and absence of the test compound (inhibitor). Double reciprocal (Lineweaver–Burk) plots were generated and used to determine the inhibition types *K*_*m*_ and *V*_max_. The inhibitory constants (*K*_*i*_) of the test compounds were also calculated.

### 2.7. Cell Culture and Maintenance

The 3T3-L1 preadipocytes and human hepatoma (HepG2) cell lines were cultured and maintained following standard protocols and procedures. Briefly, the cells were maintained and cultured in DMEM supplemented with 10% (*v*/*v*) of fetal bovine serum (FBS) and 1% (*v*/*v*) antibiotics (DMEM/FCS). Standard cell culture maintenance was followed (incubation at 37°C with 5% CO_2_ until 70%–80% confluent) with splitting at 3–4-day intervals.

### 2.8. Cell Viability Assessment

The 3T3-L1 and HepG2 cells (at 70%–80% confluence) were seeded at concentrations of 0.2 × 10^4^ per 100 *μ*L and 5.0 × 10^4^ per 100 *μ*L, respectively, in a 96-well plate. After 72 h of growth at 37°C and 5% CO_2_ until confluent, the cells were exposed to the test compounds (1–100 *μ*M) for a further 72 h at 37°C and 5% CO_2_. Control cells were exposed to either 0.01% methanol (vehicle control) or sterile phosphate buffered saline (PBS) pH 7.4. Following the exposure, the cells were fixed with 20% formalin (final concentration 2% [*v*/*v*]) at 37°C for 30 min. The solution was discarded, and the plate was blotted dry. The cells were stained with the addition of 100 *μ*L f CV (0.1% in 200 mM formic acid) to each well. After 30 min at room temperature, the dye solution was removed, and the plate was washed well with tap water and dried. The dye was then extracted from the stained cells with 100 *μ*L of a 10% (*v*/*v*) acetic acid solution. The absorbance was measured at 630 nm, and the results were expressed as percentage cell viability relative to the control (cells treated with PBS).

### 2.9. 3T3-L1 Cell Differentiation

The differentiation of 3T3-L1 cells was performed as described by Ibrahim et al. [21] with slight modifications. The preadipocyte cells were seeded at a concentration of 0.2 × 10^4^ per 100 *μ*L into each of the wells of a 96-well plate and were grown at 37°C with 5% CO_2_ for 3 days, until confluent. On Day 4, differentiation was induced by replacing the medium with 100 *μ*L Differentiation Medium 1 (DM1, DMEM supplemented with final concentrations of 10 *μ*g/mL insulin, 0.5 mM isobutyl methylxanthine, 1 *μ*M dexamethasone, and 2 *μ*M rosiglitazone) for further 3 days. On Day 8, DM1 was replaced with DM2 (DMEM with only 10 *μ*g/mL insulin) for 3 days. Finally, DM2 was replaced with only DMEM, and the cells were exposed to the different concentrations (1–100 *μ*M) of the test compounds for a further 3 days. Control cells were exposed to either the vehicle control or sterile PBS.

### 2.10. Hepatic Lipid Accumulation

The HepG2 cells were seeded in a 96-well plate at a cell density of 5 × 10^4^ and left to attach overnight. The cells were incubated at 37°C, 5% CO_2_ for 48 h, and 1 mM OA (final concentration) to stimulate lipid accumulation [22]. The cells were then treated with the drug control, lovastatin (10 and 50 *μ*M), or ARM-2 and RA-5 (10, 25, and 100 *μ*M) for a further 48 h [17]. Control cells were exposed to either the vehicle control or sterile PBS.

### 2.11. ORO Staining

The ORO staining technique was used to detect lipid droplets in both differentiated adipocytes and OA-exposed hepatocytes. The cells were fixed with 2% (*v*/*v*) formaldehyde for 30 min at 37°C. The formaldehyde was then discarded, and the plates were air-dried. The fixed cells were stained with ORO staining solution (three parts of 0.5% ORO (*w*/*v*) in 60% isopropyl alcohol and two parts of water) for 1 h. After staining, the excess ORO was discarded, and the plates were rinsed with water and dried. Microscopic images were acquired with an Olympus microscope (Olympus, Tokyo, Japan). The ORO dye was then extracted with 60% isopropanol, and the absorbance was measured at 405 nm. For the 3T3-L1 cells, the percentage of lipid accumulation was relative to the control cells (PBS-treated) cells, with 100% lipid formation, while for HepG2, the results were expressed as the percentage of lipid accumulation relative to cells exposed to OA.

### 2.12. NR Staining

With NR staining, the accumulation of neutral lipids was assessed in the differentiated 3T3-L1 cells and OA-induced lipid accumulation in HepG2 cells following treatment with the triterpenoids. The cells were washed with PBS and fixed with 4% formaldehyde for 1 h, and then the cells were stained with the NR staining solution (100 *μ*M in 100% DMSO) in the dark for 3 h. Background fluorescence was measured at an excitation and an emission wavelength of 485 nm and 590 nm (Ex_4850_ and Em_590_), respectively. The dye solution was then discarded, the wells were then rinsed with PBS, and the plate was blotted dry. The dye was extracted from the stained cells with 100 *μ*L PBS, and the fluorescence was measured again at Ex_450_ and Em_590_. The difference in fluorescence between the extracted dye and background fluorescence was calculated. The percentage of intracellular lipid accumulation relative to the untreated control cells was calculated.

### 2.13. Real-Time qRT–PCR Analysis

The mRNA expression of selected lipogenic and lipolytic genes was quantified using qRT–PCR following the established protocol as described by Ndlovu et al. [17]. In brief, total RNA was extracted from 3T3-L1 cells, which were cultured, differentiated over 10 days, and subsequently treated with 50 *μ*M simvastatin or ARM-2 or RA-5 at 25 and 100 *μ*M for 3 days. The RNA was extracted with the Promega RNA extraction kit. The RNA was then converted into cDNA using an iScript cDNA Synthesis Kit from Promega following the manufacturer's protocols. qRT–PCR analysis was performed using Bio-Rad CFX Maestro software. All mRNA expression levels were standardized to ensure consistency using glyceraldehyde 3-phosphate dehydrogenase (GAPDH) as a reference gene. The primers used were designed using the BLAST NCBI online server (https://www.ncbi.nlm.nih.gov/tools/primer-blast/). The primer sequences are given in Table 1. Data was expressed as fold change relative to the untreated control.

### 2.14. Measurement of Glycerol Release From Differentiated Adipocytes

In 96-well plates, confluent 3T3-L1 cells were induced to differentiate as described above. The cells were exposed to 50 *μ*M lovastatin or simvastatin, both control drugs, 25 and 100 *μ*M ARM-2 and RA-5, and sterile PBS and vehicle control for 3 days. Subsequently, the cells were exposed to isoproterenol (final concentration of 100 nM) for 3 h to stimulate lipolysis. The glycerol levels released into the medium were used as a measure of lipolysis evaluated using a commercial lipolysis colorimetric assay kit (MAK211) according to the manufacturer's instructions. Cell viability of the lipolysis-induced adipocytes was determined with CV staining.

### 2.15. Statistical Analysis

The experimental data are presented either as the *mean* ± *SEM* or *mean* ± *SD* of at least three independent experiments. Statistical significance was analyzed using one-way analysis of variance (ANOVA), followed by Dunnett's post hoc test using GraphPad Prism Version 7. A *p* value < 0.05 was considered to indicate statistical significance.

## 3. Results

### 3.1. Compound-Protein Docking

Virtual screening was performed to predict the binding affinities of the test triterpenoids (ARM-2 and RA-5) for several lipogenic and adipogenic proteins, namely, SREBP-1c, PPAR*γ*, C/EBP*α*, AMPK, and HSL. The docking scores are presented in Table 2. Both triterpenoids exhibited improved binding with PPAR*γ* and good binding to AMPK and SREBP-1c. Notably, although the triterpenoid Glide scores for C/EBP*α*, PNLIP, and HSL were less negative with respect to our set cut-off value of −8.00 kcal/mol, they were highly comparable to those of simvastatin and orlistat for the respective proteins (Table 2).

### 3.2. PPI

**Figure 2:**
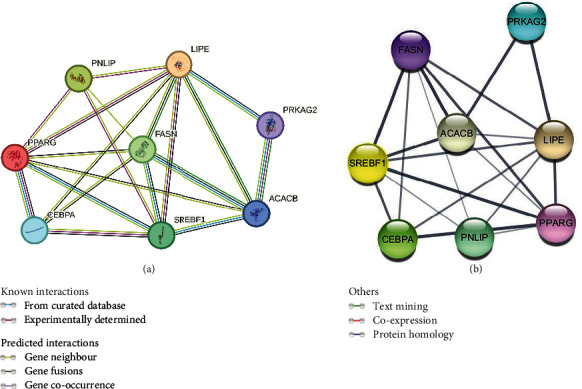
Protein–protein interactions (PPIs) of several lipogenic and lipolytic proteins. (a) The PPI network was constructed by importing eight overlapping genes and analyzing their interactions via the search tool from the Search Tool for the Retrieval of Interacting Genes/Proteins (STRING) database. (b) The associations of proteins were constructed with Cytoscape 3.10.0. The proteins are represented by nodes with gold circles, while the edges indicate associations between the proteins. Pancreatic lipase (PNLIP), hormone-sensitive lipase (LIPE), fatty acid synthase (FASN), acetyl-CoA carboxylase (ACACB), peroxisome proliferator-activated receptor-gamma (PPARG), CCAAT/enhancer-binding protein (C/EBP*α*), sterol regulatory element binding protein-1 (SREBF-1), and AMP-activated protein kinase (PRKAB1) were detected.

A PPI network of key lipogenic and lipolytic proteins (PPAR*γ*, C/EBP*α*, SREBP-1c, PNLIP, HSL, ACC, FASN, and AMPK) was constructed using the STRING database (Figure 2(a)). The nodes are interconnected by edges, with each edge representing protein–protein associations and encompassing known, predicted, and other interactions. The average node degree of freedom is 3, and the average local clustering coefficient is 0.75. Interestingly, the predictions revealed that the compounds regulate several proteins that interact with triterpenoids. Further analysis of the PPI network obtained from STRING was performed with Cytoscape software, and the degree of core expression is represented as the thickness of the edges (Figure 2(b)). The results revealed that the genes are interconnected, suggesting that the regulation of one gene may lead to the regulation of another gene. The SREBF-1 gene is coregulatory with C/EBP*α* and ACACB, and as such, its regulation and/or control could influence the expression of other genes, thereby affecting the regulation of adipogenesis and lipogenesis.

### 3.3. Inhibition of PNLIP

**Figure 3:**
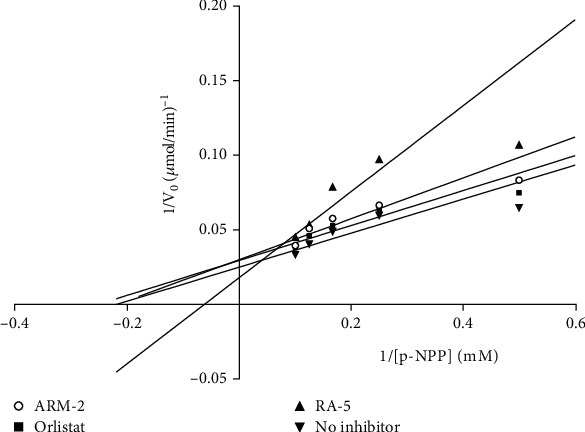
Lineweaver–Burk plot for pancreatic lipase inhibition. 1/*V*_max_: reciprocal of the maximum velocity; 1/[*S*]: reciprocal of the substrate concentration.

An in vitro assay was also used to confirm the inhibitory effect of the triterpenoids on PNLIP. The results (Table 3) revealed that both triterpenoids exhibited inhibitory effects against PNLIP, with *K*_*i*_ values of 52.86 and 28.67 *μ*M for RA-5 and ARM-2, respectively. The enzyme inhibitory activity of the compounds was further indicated by their low IC_50_ values of 27.57 and 35.83 *μ*M for RA-5 and ARM-2, respectively. However, these IC_50_ values were not comparable to those of orlistat. A Lineweaver–Burk plot (Figure 3) showed that while ARM-2 inhibits PNLIP via a noncompetitive mechanism (with the same *K*_*m*_ value), a mixed type of inhibition (with different *K*_*m*_ and *V*_max_ values) was observed for RA-5. A statistical difference between the triterpenes and orlistat was observed, further indicating the difference in the modes of action.

### 3.4. Cell Viability Assessment

**Figure 4:**
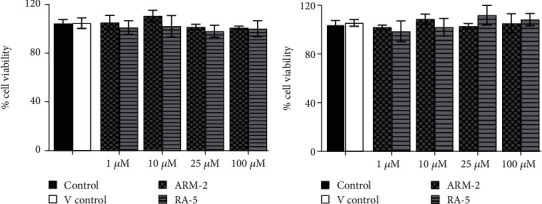
Potential cytotoxic effects of the triterpenoids on (a) 3T3-L1 and (b) HepG2 cells. The cells were exposed to ARM-2 and RA-5 for 72 h. The cells in the control group received only sterile PBS, while those in the vehicle control (*V* control) group were exposed to methanol (a carrier solvent, 0.01%). The data are expressed as the *mean* ± *SEM* of four independent experiments.

The potential cytotoxicity of the triterpenoids was investigated in both 3T3-L1 and HepG2 cells before investigating their effects on intracellular lipid accumulation. Both triterpenoids, at all the tested concentrations (1–100 *μ*M), had no cytotoxic effects on either 3T3-L1 or HepG2 cells (Figure 4).

### 3.5. Effect of Triterpenoids on Lipid Accumulation in Adipocytes

**Figure 5:**
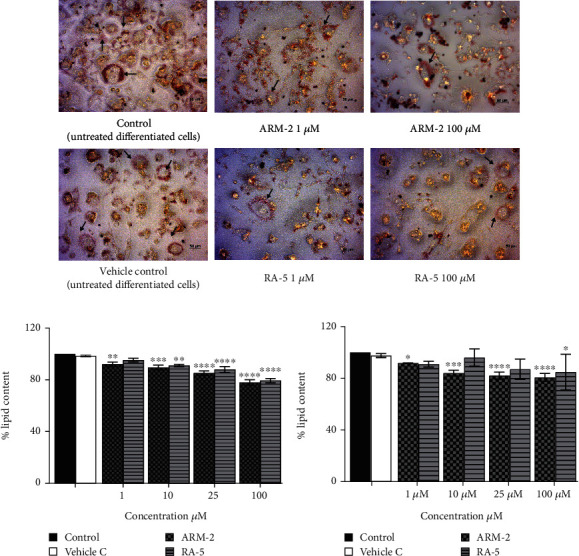
Effect of ARM-2 and RA-5 on lipid accumulation in differentiated 3T3-L1 cells. (a) ORO-stained cells and quantitative analysis of (b) intracellular lipid (ORO staining) and (c) neutral lipid (NR staining) accumulation. The cells were treated with 1, 10, 25, and 100 *μ*M ARM-2 and RA-5 for 3 days. Data was expressed as a percentage relative to the control, untreated cells (control), and as *mean* ± *SEM*; *n* = 3 independent biological repeats in triplicate; ⁣^∗^*p* < 0.05, ⁣^∗∗^*p* < 0.01, ⁣^∗∗∗^*p* < 0.001, and ⁣^∗∗∗∗^*p* < 0.0001 versus control. Scale bars are 50 *μ*m.

The effect of the triterpenoids on intracellular lipid accumulation was determined in fully differentiated adipocytes. While large intracellular lipid droplets were observed in the control cells (untreated adipocytes), treatment with both triterpenoids at 100 *μ*M reduced intracellular lipid accumulation (Figure 5(a)). A significant dosage-dependent decrease in lipid accumulation was observed for ARM-2 and RA-5. At 100 *μ*M, this was 22.21% and 20.67%, respectively (Figure 5(b)). A similar dosage-dependent decrease was observed for ARM-2 and RA-5 with a 19.27% and 10.37% reduction in neutral lipid accumulation at 100 *μ*M for ARM-2 and RA-5, respectively (Figure 5(c)).

### 3.6. Hepatic Lipid Accumulation

**Figure 6:**
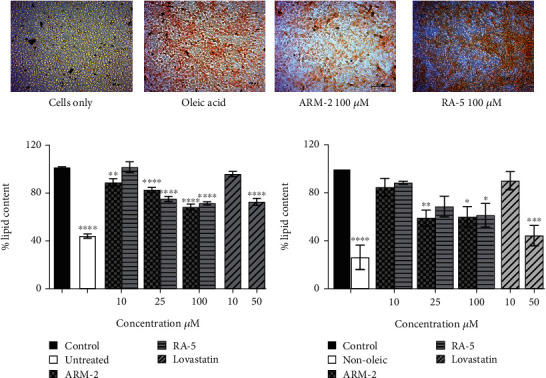
Effect of triterpenoids on oleic acid–induced lipid accumulation in HepG2 cells. (a) ORO-stained cells and quantitative analysis of (b) intracellular lipid (ORO staining) and (c) neutral lipid (NR staining) accumulation. The cells were exposed to 1 mM oleic acid for 48 h in the presence (10, 25, and 100 *μ*M) or absence of the test compounds. The untreated cells were not exposed to oleic acid. Lovastatin (50 *μ*M) was used as a positive control. The values are expressed as the *means* ± *SEMs*; *n* = 3 independent biological replicates in triplicate; ⁣^∗^*p* < 0.05, ⁣^∗∗^*p* < 0.01, ⁣^∗∗∗^*p* < 0.001, and ⁣^∗∗∗∗^*p* < 0.0001 versus the control. The scale of the images is 50 *μ*m.

The potential effect of ARM-2 and RA-5 on intracellular lipid accumulation was also determined in the HepG2 cell model of OA-induced hepatic lipid accumulation. Exposure to OA caused an increase in lipid accumulation in HepG2 cells (Figure 6(a)). Treatment with 100 *μ*M ARM-2 or RA-5 reduced the lipid accumulation, as indicated by fewer lipid droplets following ORO staining (Figure 6(a)). Quantification revealed a statistically significant dosage-dependent reduction, with a reduction of 31.56% and 23.47% after treatment with 100 *μ*M ARM-2 and RA-5, respectively. A dose–response reduction in the hepatic lipid accumulation was also observed, which was significant for ARM-2 (10–100 *μ*M) and RA-5 (25 and 100 *μ*M) (Figure 6(b)). Neutral lipid accumulation was only significant for ARM-2 (25 and 100 *μ*M) and RA-5 (100 *μ*M) with a 40% and 39% reduction in neutral lipid accumulation for 100 *μ*M ARM-2 and RA-5, respectively (Figure 6(c)). Likewise, as in differentiated 3T3-L1 cells, the effect on OA-induced lipid accumulation in HepG2 cells was greater for ARM-2 than for RA-5. The effect of 50 *μ*M of the control, lovastatin, was similar to the effect of 25 and 100 *μ*M ARM-2 and RA-5.

### 3.7. Expression of Several Lipolytic and Lipogenic Genes

**Figure 7:**
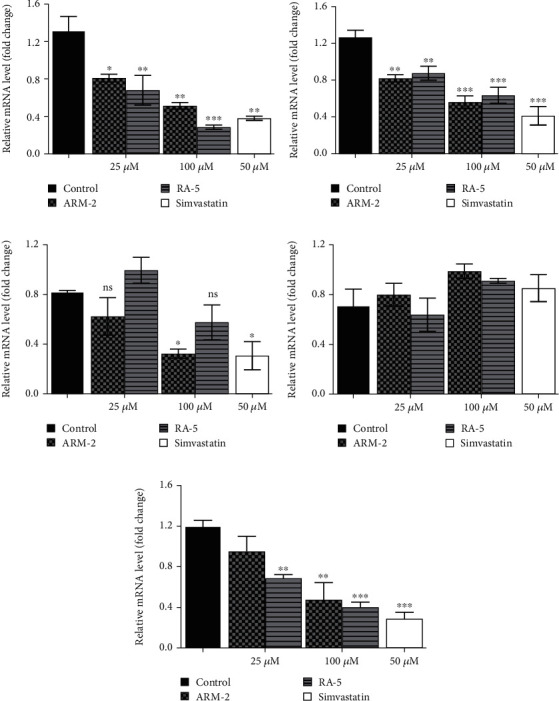
Effects of the triterpenoids on several selected lipolytic, lipogenic, and adipogenic genes. The mRNA levels of (a) PPAR*γ*, (b) C/EBP*α*, (c) SREBP-1, (d) AMPK, and (e) HSL were quantified in differentiated 3T3-L1 cells. Simvastatin was used as a positive control. The data are expressed as the *mean* ± *SD* of duplicate samples. ⁣^∗^*p* < 0.05, ⁣^∗∗^*p* < 0.01, and ⁣^∗∗∗^*p* < 0.001 versus the control.

RT-qPCR was used to quantitatively determine the effect of the triterpenoids on some selected lipolytic, lipogenic, and adipogenic genes in differentiated 3T3-L1 adipocytes. Increased expression of mRNA levels on PPAR*γ*, C/EBP*α*, SREBP-1, and HSL, along with reduced expression of AMPK mRNA levels, was observed in the control, untreated differentiated cells (Figure 7). Interestingly, treatment of the cells with ARM-2 and RA-5, at 100 *μ*M, significantly reduced the expression of PPAR*γ* (59.30% and 77.40%), C/EBP*α* (55.65% and 49.72%), SREBP-1 (59.30% and 77.40%), and HSL (60.38% and 66.54%) genes (Figures 7(a), 7(b), 7(c), and 7(e)) which translates into a significant fold decrease in the relative mRNA levels. An increase in the expression of the *AMPK* gene of 39.10% and 28.05% was also observed following the treatment with ARM-2 and RA-5, respectively (Figure 7(d)), although not significant when compared with the control. It is noteworthy that ARM-2 demonstrated high efficacy in comparison to simvastatin in regulating AMPK. No significant differences were observed between 100 *μ*M ARM-2 and RA-5 and 50 *μ*M simvastatin, the drug control. This indicates that for all genes evaluated, ARM-2 and RA-5 downregulate lipolytic and lipogenic genes similar to simvastatin.

### 3.8. The Effect of Triterpenoids on Lipolysis in 3T3-L1 Cells

**Figure 8:**
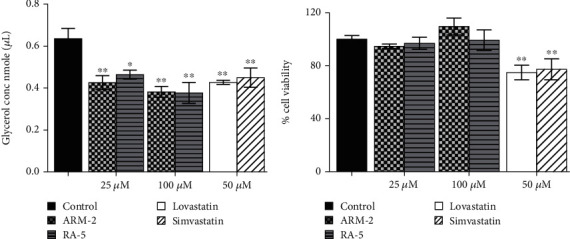
Effect of triterpenoids on lipolysis in 3T3-L1 cells. The differentiated cells were exposed to isoproterenol for 3 h to stimulate lipolysis in the presence or absence of triterpenoids at 25 and 100 *μ*M. (a) The content of glycerol released by differentiated 3T3-L1 cells and (b) the percentage viability of the cells after exposure to the test compounds for 3 days. The values are expressed as *mean* ± *SEMs*; *n* = 3 experiments performed in triplicate; ⁣^∗^*p* < 0.05 and ⁣^∗∗^*p* < 0.01 versus the control.

A glycerol release assay was also used to investigate the effect of triterpenoids (ARM-2 and RA-5) on lipolysis in 3T3-L1 cells (Figure 8). While a higher glycerol content was detected in the control untreated cells, a decrease in glycerol levels was observed in the triterpenoid-treated cells. At 100 *μ*M, ARM-2 and RA-5 decreased glycerol release by 36.47% and 34.72%, respectively (Figure 8(a)). While the triterpenoids did not have cytotoxic effects on lipolysis-stimulated differentiated adipocytes, both simvastatin and lovastatin at 50 *μ*M had cytotoxic effects; thus, the observed reduction in glycerol release could also be related to cell death (Figure 8(b)).

## 4. Discussion

The clinical limitations linked to the currently used hypolipidemic drugs continue to fuel the search and discovery of new alternative drugs. Medicinal plants are considered valuable sources of potential lead molecules as new blood lipid–lowering drugs with multiple targets, improved efficacy, and fewer side effects. In this endeavour, our research group has recently reported the hypocholesterolemic effect of the two triterpenoids (ARM-2 and RA-5) from *P. longifolia* [17]. The identified bioactivities were linked to the regulation of dietary cholesterol absorption and biosynthesis and stimulation of cellular LDL uptake. This study was aimed at exploring further the hypolipidemic potential of ARM-2 and RA-5 using in silico and in vitro cell models.

Dietary TGs contribute significantly to the elevation of circulating TG levels in the bloodstream and are primarily due to the uncontrolled digestion and absorption of TG in the intestinal lumen following consumption of TG-rich diets. Hence, a primary strategy to reduce blood TG levels is to delay dietary TG digestion and absorption by inhibiting PNLIP [23]. The inhibitory activity of the triterpenoids, RA-5 and ARM-2, with varying levels of efficacy compared with orlistat on PNLIP identifies some potential to inhibit dietary TG digestion, thereby delaying TG absorption. In silico predictions indicated that both ARM-2 and RA-5 exhibit a similar competitive mode of inhibition to orlistat; however, in vitro enzyme inhibition studies identify different modes of inhibition. The noncompetitive and mixed types of inhibition displayed by ARM-2 and RA-5, respectively, indicate that inhibition is mediated by an interaction with the allosteric site of PNLIP.

Elevated blood levels and the subsequent accumulation of TG in matured adipocytes are closely associated with obesity, which is strongly linked to the global health threat of metabolic syndrome [24]. In vitro studies showed that ARM-2 and RA-5 treatment reduced the lipid droplet accumulation (Figures 5(a) and 6(a)), total lipids (Figures 5(b) and 6(b)) and neutral lipid accumulation (Figures 5(c) and 6(c)) in differentiated 3T3-L1 adipocytes (Figure 5) and the OA-induced HepG2 cell model (Figure 6). In the latter model, cellular lipid–lowering effects were similar to lovastatin. To address obesity and its related complications, AMPK emerges as a valuable therapeutic target due to its role in maintaining energy homeostasis [25]. In this process, AMPK suppresses the expression of SREBP-1, the transcriptional factor that controls the expression of lipogenic genes such as fatty synthase (FAS), ACC, PPAR*γ*, and C/EBP*α*. The suppression of these genes inhibits lipogenesis [25–27].

In this study, AMPK expression was unaltered while the expression of SREBP-1, PPAR*γ*, and C/EBP*α* genes after treatment of differentiated 3T3-L1 adipocytes with ARM-2 and RA-5 was reduced (Figure 7). The pattern of unaltered AMPK expression and reduced expression of the SREBP-1, PPAR*γ*, and C/EBP*α* genes is similar to the effect of simvastatin evaluated within the same concentration range. This indicates that the antilipogenic effect of the triterpenoids similar to simvastatin is linked to the activation of the AMPK/SREBP-1 pathway. Interestingly, the in silico results predicted that both triterpenoids have a favourable binding on SREBP-1 and AMPK indicating a possible direct interaction with their binding sites. Moreover, the predicted PPI network (Figure 2) confirms that the expression of SREBP-1 can directly influence the expression levels of FAS, ACC, and C/EBP*α*, thus modulating lipogenesis.

Beyond the role of triterpenoids regulating lipogenesis, the triterpenoids have also exhibited the ability to attenuate adipogenesis. The predicted interactions and ability to reduce the expression of key genes such as PPAR*γ* and C/EBP*α* potentially lead to reduced adipocyte hyperplasia and hypertrophy. Although there is limited evidence regarding the antilipogenic effect of statins, previous studies have identified that simvastatin has antiadipogenic effects by suppressing PPAR*γ* expression [28, 29] and potential antilipogenic effects by modulating the AMPK pathway [27].

In response to high energy demands, TGs stored in adipose tissue undergo lipolysis, a catabolic pathway that promotes the mobilization of metabolic fuel from adipose to peripheral tissues [30]. Dysregulated lipolysis, often associated with increased HSL activity, can lead to an increased flux of nonesterified fatty acids (FFAs) and glycerol into the blood circulation. The reduction in glycerol content released during the isoproterenol-stimulated lipolysis in the differentiated 3T3-L1 adipocytes treated with the triterpenoids (Figure 8(a)) revealed the antilipolytic effect of the compounds similar to lovastatin and simvastatin. Isoproterenol, a synthetic catecholamine, triggers lipolysis by activating the cyclic AMP (cAMP)–dependent pathway, which in turn leads to HSL phosphorylation [31]. Despite both triterpenoids demonstrating less favourable binding affinity on the catalytic site of HSL in silico, the observed decrease in mRNA levels of the *HSL* gene in differentiated adipocytes treated with the compounds further supports the potential regulation of lipolysis. The observed antilipolytic properties of ARM-2 and RA-5 are consistent with literature reports on other plant-derived triterpenoids that suppress HSL expression [32, 33]. While experimental evidence suggests that simvastatin administration promotes lipolysis via HSL phosphorylation [34, 35], our findings indicate that simvastatin potentially controls lipolysis by decreasing HSL expression. Atorvastatin, a costatin, has previously been reported to control lipolysis by decreasing the expression of HSL [36].

Furthermore, the flux of FFA, mainly from adipocytes, into the blood stimulates hepatic fatty acid uptake and subsequent de novo lipogenesis. The development of HTG is largely related to excessive hepatic synthesis and secretion of VLDLs and impaired plasma TRL clearance [37]. Interestingly, the observed decrease in intracellular lipid accumulation in the HepG2 cells following their treatment with the triterpenoids (Figure 6) indicated the potential of ARM-2 and RA-5 to regulate plasma TG levels by controlling hepatic TG production and release. Treatment with plant-derived triterpene, astragaloside, and ginsenoside CK reduced hepatic lipid accumulation in the OA-induced hepatic lipid accumulation model using the HepG2 cells [38, 39].

## 5. Conclusion

The regulation of both lipogenesis and lipolysis is crucial in maintaining energy homeostasis and actively managing circulating TG and FFA levels. The results obtained from the present study revealed the antilipogenic and antilipolytic effects of ARM-2 and RA-5. The antilipogenic activity of both triterpenoids is related to the inhibition of fatty acid synthesis, which results in a decrease in hepatic and adipocyte lipid accumulation, with similar effects on SREBP-1c, PPAR*γ*, C/EBP*α*, and AMPK gene expression and possibly mediated via the AMPK/SREBP-1c pathway. The inhibition of adipocyte lipolysis was also similar to known statins, lovastatin and is simvastatin, and potentially due to the downregulation of HSL. The obtained results suggest that the triterpenoids, ARM-2 and RA-5, have the potential to modulate lipogenesis and lipolysis. Furthermore, Western blot analysis to confirm gene expression findings at the protein level, along with the quantification of FFA release, the phosphorylation status of HSL, is recommended fo future studies. Investigation of the effects of the triterpenoids on ATGL and FASN, and intracellular cAMP levels, is also recommended for future studies, as these were limitations of the current study. Additionally, more in-depth mechanistic studies, both in vitro and in vivo, are suggested for future investigations to provide a comprehensive understanding of the antihypertriglyceridemic potential and underlying mechanisms of action of ARM-2 and RA-5.

## Figures and Tables

**Table 1:** Primer sequences of lipolysis and lipogenesis.

**Gene**	**Forward (5 **⁣′**-3 **⁣′**)**	**Reverse (5 **⁣′**-3 **⁣′**)**
GAPDH	TCCTGTTCGACAGTCAGCCG	CCCCATGGTGTCTGAGCGAT
PPAR*γ*	CTCGAGGACACCGGAGAGGG	GCATCCGCCCAAACCTGATG
HSL	GGAACGAATCTGCCTTGCGG	CGGAAGTCTCGGAGGAGCAG
AMPK	CGGCAAAGTGAAGGTTGGCA	GGGCCGAGTCAGGTGATGAT
C/EBP*α*	GTGCGAGCCAGGACTAGGAG	GCTGTAGCCTCGGGAAGGAG
SREBP-1c	TTCCGAGGAACTTTTCGCCG	CTGCTGGATCTGCGAGGTCA

Abbreviations: AMPK, activated mitogen protein kinase; C/EBP*α*, CCAAT/enhancer-binding protein; HSL, hormone-sensitive lipase; PPAR*γ*, peroxisome proliferator-activated receptor *γ*; SREBP-1, sterol regulatory element binding transcription factor 1.

**Table 2:** XP Glide scores of ARM-2, RA-5, orlistat, and simvastatin for HSL (3DNM), PL (2PPL), AMPK (5ISO, 4CFF), SREBP-1 (5GPD), PPAR*γ* (5GTN), and C/EBP*α* (1NWQ).

**Ligand**	**PL**	**HSL**	**AMPK**	**SREBP-1**	**PPAR*γ***	**C/EBP*α***
ARM-2	−4.228	−3.160	−5.479	−7.150	−8.999	−4.270
RA-5	−2.927	−4.261	−6.041	−7.621	−8.952	−3.864
Orlistat	−4.187	−4.286	−3.810	−9.632	−12.121	−3.032
Simvastatin	−4.663	−4.203	−6.613	−8.624	−10.433	−5.174

**Table 3:** The inhibitory effects of the tested triterpenoids on pancreatic lipase activity, as determined by the IC_50_, *K*_*m*_, *V*_max_, and *K*_*i*_.

**Inhibitor**	**Pancreatic lipase**				
	**IC **_**50**_ ** (*μ*M)**	***K***_***i***_ ** (*μ*M)**	***K***_***m***_ ** (*μ*M)**	***V***_**max**_ ** (*μ*M)**	**Mode of inhibition**
ARM-2	35.83 ± 12.44^∗^	52.86 ± 7.19	1.18 ± 0.35	3.50 ± 1.10	Noncompetitive
RA-5	27.57 ± 8.51^∗^	28.67 ± 8.84	1.91 ± 1.45	4.64 ± 2.98	Mixed
Orlistat	3.12 × 10^−2^ ± 2.35 × 10^−3^	19.18 ± 1.28	5.2 ± 0.19	1.66 ± 0.86	Uncompetitive
No inhibitor			2.14 ± 1.00	1.43 ± 0.051	

*Note:* Data are expressed as the *mean* ± *SEM* (*n* = 3).

⁣^∗^*p* < 0.0001 versus orlistat.

## Data Availability

The data used to support the findings of this study are included within the article.
